# Energy Equivalence Based Estimation of Hybrid Composites Mechanical Properties

**DOI:** 10.3390/ma16124215

**Published:** 2023-06-06

**Authors:** Anna Jerzyńska, Halina Egner

**Affiliations:** Department of Applied Mechanics and Biomechanics, Faculty of Mechanical Engineering, Tadeusz Kosciuszko Cracow University of Technology, 37 Jana Pawla II Av., 31-864 Cracow, Poland

**Keywords:** isotropic composites, inclusions, hybrid structures, elastic properties estimation

## Abstract

Hybrid composites, usually combining natural and synthetic reinforcing filaments, have gained a lot of attention due to their better properties than traditional two-component materials. For structural applications of hybrid composites, there is a need to precisely determine their mechanical properties on the basis of the mechanical properties, volume fractions, and geometrical distributions of constituent materials. The most common methods, such as the rule of mixture, are inaccurate. More advanced methods, giving better results in the case of classic composites, are difficult to apply in the case of several types of reinforcement. In the present research, a new estimation method is considered, which is simple and accurate. The approach is based on the definition of two configurations: the real, heterogeneous, multi-phase hybrid composite configuration, and the fictitious, quasi-homogeneous one, in which the inclusions are “smeared out” over a representative volume. A hypothesis of the internal strain energy equivalence between the two configurations is formulated. The effect of reinforcing inclusions on the mechanical properties of a matrix material is expressed by functions of constituent properties, their volume fractions, and geometrical distribution. The analytical formulas are derived for an isotropic case of a hybrid composite reinforced with randomly distributed particles. The validation of the proposed approach is performed by comparing the estimated hybrid composite properties with the results of other methods, and with experimental data available in the literature. It is shown that a very good agreement is obtained between experimentally measured hybrid composite properties and their predictions resulting from the proposed estimation method. The estimation errors are much lower than the errors of other methods.

## 1. Introduction

Hybrid composites are materials that consist of a matrix and at least two different types of reinforcement. They can be manufactured from a combination of a variety of organic or inorganic materials such as metals, ceramics, polymers, etc., to obtain the desired mechanical, thermal, electrical, or other properties. Virtually unlimited possibilities of combining several organic or inorganic constituents allow the designing of a huge number of new products with high precision, as well as modifying conventional composite materials.

Hybrid composites usually exhibit properties that neither organic nor inorganic constituent materials can reach separately; they may be flexible and at the same time be characterized by high mechanical strength and thermal stability. Moreover, the resulting properties of the combination of several reinforcing materials may exhibit a synergistic effect [1]. For example, composites often have better fatigue properties than metals [2]. Although initial fatigue damage may appear earlier in composites than in metals, further damage development may slow down due to arresting microcracks in the internal structure of a composite, while in metals cracks usually rapidly develop [3].

Increasing demand for eco-friendly materials encourages implementing agricultural waste as natural fibers, increasingly used as an addition or replacement to traditional synthetic fibers, which have harmful effects on the environment. For this reason, the hybrid use of natural and synthetic reinforcing materials has gained a considerable attention from researchers [4,5]. Generally, synthetic fibers exhibit better mechanical properties, while natural fibers, despite their weaker mechanical properties, may have a positive effect e.g., on reducing the weight of the composite, which is a feature desirable especially for lightweight structures. One of the sectors for which weight reduction is important is the automotive industry. For example, the combination of kevlar and date palm fibers gives the composite better enhancement in tensile strength and tensile modulus. Such dependence has been observed by Muthalagu et al. [6] who studied the mechanical properties of kevlar fiber/date palm fiber/epoxy resin hybrid composite in the context of the application for passenger car bumper beams. Composites with different weight percentages of reinforcements were fabricated by hand lay-up method and tested in a tensile test. A bumper model was also performed by CATIA and subjected to a finite element analysis in ANSYS. Ganesarajan et al. [7] provide a comprehensive study on the development of hybrid composites containing engineered poly-saccharide to deliver optimum mechanical properties suited for body interior and under-the-hood applications for passenger vehicles or light-duty trucks. The proposed solutions exhibit progress in light-weighting, manufacturing cycle time, and overall material sustainability. In paper [8] natural rubber toughened silica/kenaf fiber/epoxy resin composites are investigated for possible automotive applications. The addition of liquid rubber MG30 produced rubber particles in the composite helped to increase the overall mechanical properties. Mansor et al. [9] used the Analytical Hierarchy Process (AHP) method to select the most proper natural fiber for hybridization with glass fiber reinforced polymer composites for the design of a passenger vehicle brake lever. Of the 13 fiber types tested, kenaf fiber turned out to be the best option which fulfills performance requirements and the design objectives. Dattatreya et al. [10] studied the mechanical properties of hybrid composites made of waste natural fibers or fillers and epoxy resin. The research included composites with jute fiber, coconut coir, sugarcane, and neem wood powder. Due to its high tensile properties, jute fiber was chosen as the base material and the other three fillers of hybrid composites had different weight percentages. The composite composed of 15% neem wood waste, 5% coconut fiber, and 5% sugarcane bagasse achieved the best results in terms of flexural, tensile, and impact strength. Which makes these composites potentially useful in the automotive industry.

Considering other fields, Bagheri et al. [11] investigated carbon fiber/flax fiber/epoxy resin hybrid composite for bone implants. Compared to commonly used metal plates for long bone fracture treatment, the mechanical properties of the hybrid composite were closer to human cortical bone. The presented results suggest that this material may be considered for the use in long bone fracture fixation. Scutaru and Baba [12] studied the impact properties of carbon fiber/hemp fiber/polyester resin laminates. They concluded a high stiffness of investigated hybrid composite. Additionally, carbon fiber/hemp fiber composite laminates have good thermal properties. Due to this, they are used e.g., for insulators in spaceships, airplanes, and nuclear reactors. Cicala et al. [13] tested the possibility of using glass fiber/natural fiber/epoxy resin hybrid composites for applications in the piping industry. Hemp, kenaf, and flax fiber were used. Manufactured hybrid composites allowed for a reduction in cost by 20% and a reduction in weight by 23% compared to the current commercial solutions. Hybrid composites are also used on boat components such as propellers and masts. Research on one of the first prototypes of a glass fiber/carbon fiber hybrid composite mast revealed improvements in ballistic performance as well as stiffness. A weight reduction of 20–50% compared to an aluminum shaft of the same size has also been reported [14].

Panthapulakkal and Sain [15] investigated water absorption and mechanical and thermal properties of glass fiber/hemp fiber/polypropylene hybrid composites. Results show that the addition of glass fibers to hemp fiber/polypropylene composite improved all studied material properties and that such a hybrid material can be successfully used for structural applications where thermal resistance and high stiffness are desired.

Following the expanding application of hybrid composites, it is necessary to extend the use of classical averaging methods for two-component composite properties to hybrid composites with a larger number (in general: *n*) of reinforcing materials. The simplest classical models for the estimation of the average elastic moduli of a two-component composite are the Voigt [16] and Reuss [17] mixture rules based on volume fractions of component materials, and are proved (cf Hill [18]) to constitute respectively the upper and lower bounds of the real effective stiffness values. However, neither the Reuss nor the Voigt assumptions are exact. For the Voigt approach, the tractions and phase boundaries may not be in equilibrium, while for the implied Reuss strains the matrix and inclusions may not remain bonded. Additionally, the mentioned bounds are usually distant from each other, therefore there is a wide range of possible predicted values of the effective elastic properties. Hashin and Shtrikman [19] used the concept of polarization and the principle of minimum potential energy to provide narrower bounds for isotropic composites. Another popular homogenization method named the Self-Consistent Scheme (SCS) [20] assumes that the single particle is embedded in an effective medium of unknown properties. This method provides the implicit expressions for the effective shear and bulk moduli. A different concept is due to Nazarenko et al. [21], who considered the effective properties of composites with the interphases characterized by the surface-varying properties. Many classical theories and homogenization methods account both for the topology of reinforcement and the material symmetry of a constitutive model describing a composite (cf. for ex. Sun and Vaidya [22], Gan et al. [23], Liu et al. [24], Würkner et al. [25], Selvadurai and Nikopour [26] and others). Generally, it is assumed that the distribution of component materials has a periodic manner, therefore it is possible to assume the existence of the so-called representative unit cell (RUC).

Predicting the effective properties of hybrid composites becomes more complicated with a growing number of components, and so far, there has been little theoretical modeling of property relationships in these materials. In [27] the classical lamination approach to predict the theoretical elastic modulus of a natural fiber-reinforced hybrid composite is used. It is shown that the theoretical values of elastic and flexural moduli were not accurately estimated by the hybrid mixture method. The application of the Mori–Tanaka method [28] or self-consistent method to the solution of this problem gives overall elastic moduli tensors of such composites that do not exhibit the necessary symmetry [29]. The hybrid graphene/carbon nanotube (CNT)/elastomer nanocomposite is considered in [30] as a pressure sensor under bending. Both graphene and CNTs are assumed to be randomly distributed in the composite and treated as ellipsoidal inclusions in an isotropic matrix. The effective elastic modulus was estimated by the use of the Mori–Tanaka method and compared to the experimental data.

In the majority of research papers, multi-step homogenization is applied. Hasanzadeh et al. [31] propose a multi-procedure micromechanics approach based on the Mori–Tanaka model to obtain the properties of piezoelectric hybrid composites containing carbon nanotubes. In the first step, the elastic properties of a nanocomposite consisting of randomly distributed CNTs in the polymer matrix are modeled. In the second step, considering the nanocomposite as a matrix and piezoelectric fiber as reinforcement, the elastic properties of CNT–piezoelectric fiber-reinforced hybrid composites are predicted. Bazan et al. [32] proposed a two-step homogenization procedure to predict the effective Young modulus of the three-phase short-fiber-reinforced composite. In the first step, the estimation of the effective elastic modulus is performed using the Halpin-Tsai [33] approximation, applied to the matrix and one of the reinforcing components. In the second step, the property estimated in the first step is used as the “matrix” modulus and homogenized with the second reinforcing phase. In paper [34] a multiscale finite element technique and a sequential methodology are used to estimate the mechanical properties of hybrid composites made of a polymer matrix, reinforced with carbon fibers and silica microparticles. The results show a very good agreement with the experimental data.

Although the classical Voigt’s rule of mixture was shown to result in significant discrepancies with the experimental results, especially in the case of natural fibers [35], it can still be found in use due to its simplicity [36,37].

The approach to predict the elastic properties of a hybrid composite presented in this research utilizes a concept of a fictitious quasi-homogeneous continuum [38], in which the inclusions are “smeared out” within a representative volume element (RVE). The hypothesis of equivalence between the fictitious quasi-homogeneous continuum and the real composite material configuration is then postulated. The approach is analogous to the formalism of the continuum damage mechanics (CDM), which deals with materials containing microdefects that degrade the material properties. The subject of the current approach is a material with inclusions that improve its properties, which is the opposite effect; nevertheless, it can be properly reflected with the use of the same thermodynamic formalism.

The first attempts to extend the CDM-based approach to a general case of thermodynamically based constitutive modeling were performed by Egner and Ryś [39,40,41]. The idea to apply such reasoning to the composite material properties estimation was started in the paper by Wiśniewska et al. [42], where the basic concept of mapping between a real composite and a fictitious quasi-homogeneous configuration was formulated and used to estimate elastic properties of isotropic two-component composite materials. The approach was furtherly developed by Wiśniewska and Egner in [38]. Different forms of an inclusion-effect tensor were discussed in terms of its influence on the stress and strain states in a fictitious quasi-homogeneous configuration. Additionally, the concentration factors were derived and compared with other homogenization methods, and an optimization problem of an FGM shaft was analyzed with the use of the proposed estimation method. The extension of the method for inelastic material properties of two-component isotropic composites was presented in [43].

In the present paper, this concept is further developed and applied for the first time to hybrid composite materials. A significant advantage of the method used in the presented research in comparison with the above-mentioned classical approaches is the simplicity of calculations and the possibility to predict with significantly better accuracy the effective properties of composite materials. What is more, there is no need to incorporate any multi-step procedures.

## 2. Theoretical Formulation

### 2.1. Basic Ideas

The main assumption adopted in the considered homogenization method states that the effective properties of an isotropic composite can be described by volume averaging of the actual properties of its components (matrix and reinforcing inclusions) over a domain called the representative volume element (RVE) which maps a finite volume of the real heterogeneous hybrid composite material into a material point of the fictitious pseudo-homogeneous configuration. This effective quasi-homogeneous configuration method is based on the following assumptions:

- Each inclusion within the RVE matrix is subjected to the same stress field derived from the external tractions applied at the boundary of this element.
- The effect of all other inclusions within the RVE matrix on the observed inclusion is measured through the change of effective composite stiffness or compliance.
- For the isotropic short fiber-reinforced composites the exact spatial correlation of the inclusions within the RVE has negligible influence on the effective composite properties defined within the element.

A transition between the microscale and the effective composite properties on the macroscale requires a proper selection of the RVE size, bounded by two constraints. On one hand, it must be large enough to include a sufficient number of reinforcing inclusions that justify homogenization within the RVE. However, on the other hand, the size of the RVE must be small enough for the stress state to be considered homogeneous. The existence of the RVE, which allows the material to be considered statistically homogeneous within the volume element, is the condition for the presented approach, in which there are no scale parameters involved. The true distribution of the inclusions within the RVE matrix, described by the (scalar or tensorial) parameter ξ, results in the change of the macroscopic constitutive properties of a composite, which may be estimated on the basis of the mechanical equivalence hypothesis. From among several physical quantities (strain, stress, elastic strain energy, etc.), the equivalence of the total strain energy between the real composite configuration and the fictitious quasi-homogeneous configuration is here assumed, which allows determining not only elastic mechanical properties but also inelastic parameters [43].

The total strain energy equivalence (TEE) hypothesis can be described as follows [38,39,42,43,44]:

At any time, to an RVE in its real (multiphase, hybrid) configuration, described by the set of state variable pairs $V_{\alpha }$ (stresses, strains), we associate a homogeneous (monophase) equivalent fictive configuration, the state of which is described by the effective state variables ${\tilde{V}}_{\alpha }$ (effective stresses, effective strains)—in such a manner that the total internal strain energy U defined over the two (real and fictive) configurations is the same:

(1) $$U\left(V_{\alpha },\xi \right)=U\left({\tilde{V}}_{\alpha },0\right)\;$$

**Figure 1:**
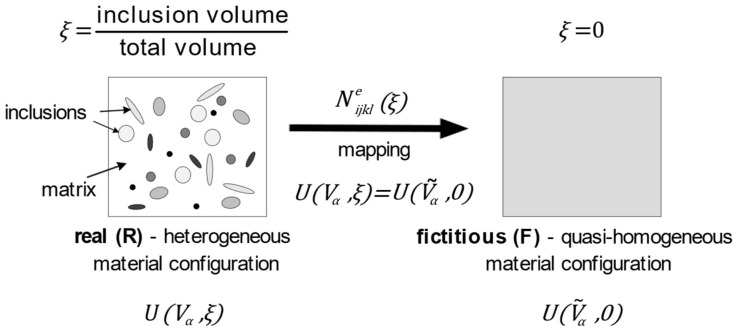
Schematic representation of energy equivalence idea between real (R) and fictitious (F) configurations.

Accordingly, the definitions of effective stresses ${\tilde{\sigma }}_{ij}$ and effective elastic strains ${\tilde{\varepsilon }}_{ij}^{e}$ are related to the two above mentioned configurations: real (R) and fictitious (F). Assuming a linear relation between two second-order symmetric tensors, the relation between the variables $V_{\alpha }$ and ${\tilde{V}}_{\alpha }$ may be expressed in the most general form with the use of the fourth-order tensor $N_{ijkl}^{e}$, called here the inclusion-effect tensor. This tensor allows mapping from heterogeneous (R) to quasi-homogeneous (F) configuration (cf [45,46,47], see Figure 1):

(2) $${\tilde{\sigma }}_{ij}={\left[N_{ijkl}^{e}\left(\xi \right)\right]}^{-1}\sigma_{kl},\;{\tilde{\varepsilon }}_{ij}^{e}={\left[N_{ijkl}^{e}\left(\xi \right)\right]}^{T}\varepsilon_{kl}^{e}$$

In the simplest case of an isotropic composite with randomly distributed and oriented short reinforcing fibers, the inclusion-effect tensor was proposed in [42] in the following isotropic two-parameter form:

(3) $$N_{ijkl}^{e}\left(\xi \right)=f_{1}\left(\xi \right)\delta_{ij}\delta_{kl}+f_{2}\left(\xi \right)\left(\delta_{ik}\delta_{jl}+\delta_{il}\delta_{jk}\right),$$

where $f_{1}\left(\xi \right)$, $f_{2}\left(\xi \right)$ are scalar functions of the parameter ξ, defined in the isotropic case as the reinforcement volume fraction $dV^{I}$ in the total volume $dV^{RVE}$ of the RVE:

(4) $$\xi =\frac{dV^{I}}{dV^{RVE}}$$

By the use of the total strain energy equivalence hypothesis, the elastic stiffness tensor $E_{ijkl}\left(\xi \right)$ of a composite material results from the equivalence between elastic strain energies:

(5) $$U^{e}=\frac{1}{2}\sigma_{ij}\varepsilon_{ij}^{e}=\frac{1}{2}{\tilde{\sigma }}_{ij}{\tilde{\varepsilon }}_{ij}^{e},$$

Taking into account Equations (2) and (5) and the Hooke law, the elastic stiffness tensor $E_{ijkl}\left(\xi \right)$ takes the following form, dependent on the matrix stiffness tensor $E_{ijkl}^{M}\left(\xi \right)$ and the inclusions volume fraction ξ [38,42,43]:

(6) $$E_{ijkl}\left(\xi \right)=N_{ijpq}\left(\xi \right)E_{pqrs}^{M}N_{rskl}\left(\xi \right),$$

The above equation can be transformed with the use of Equation (3) into the following form:

(7) $$E_{ijkl}\left(\xi \right)=\lambda \left(\xi \right)\delta_{ij}\delta_{kl}+\mu \left(\xi \right)\left(\delta_{ik}\delta_{jl}+\delta_{il}\delta_{jk}\right),$$

where $\lambda \left(\xi \right)$ and $\mu \left(\xi \right)$ are the effective Lamé constants dependent on the matrix constants $\lambda^{M}$, $\mu^{M}$, and the volume fraction of inclusions:

(8) $$\lambda \left(\xi \right)=\lambda^{M}\left[9f_{1}^{2}+12f_{1}f_{2}+4f_{2}^{2}\right]+2\mu^{M}\left[3f_{1}^{2}+4f_{1}f_{2}\right],$$

(9) $$\mu \left(\xi \right)=4\mu^{M}f_{2}^{2}.$$

The details of the above transformations can be found in [42], and will not be repeated here.

**Figure 2:**
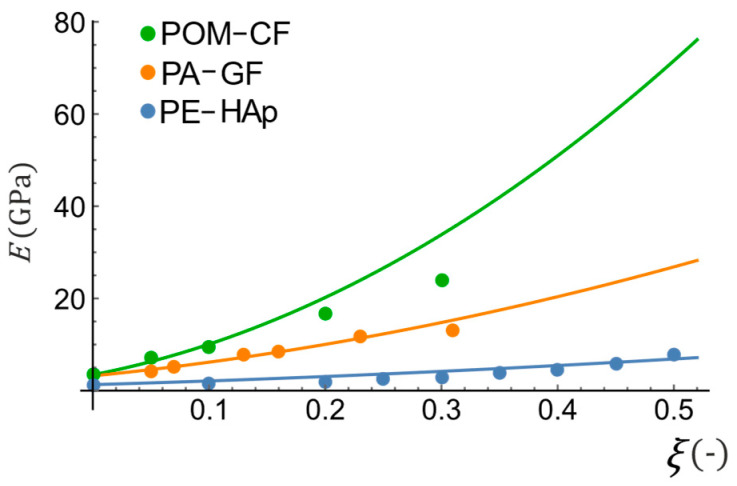
Comparison between the theoretical estimations of composite elastic properties and experimental data. Data adapted from [48,49,50].

The elastic properties estimation based on the hypothesis of total strain energy equivalence allows obtaining a very good agreement with the experimental data, while the calculations are relatively simple. Figure 2 shows several examples of composite material properties estimations compared with the experimental data. In this graph, solid lines represent total strain energy equivalence-based (TEE) estimations, while dots stand for the experimental results of the Young modulus for carbon short-fiber reinforced polyacetal (POM-CF), glass short-fiber reinforced polyamide (PA-GF) [48], and hydroxyapatite reinforced polyethylene (PE-HAp) [49,50]. 

There are some small discrepancies for the POM-CF composite, while for the other two materials, the predictions are excellent. It should be pointed out that the method proposed here, like all other homogenization methods, is based on simplifying assumptions and therefore approximate (for example, the matrix/inclusion interphase interaction is disregarded). Nevertheless, the errors in predicted values are very small in comparison with other classical methods. As it can be seen in Table 1, for more than 60% of results the difference between experimental and predicted Young modulus values, related to the experimental value (error), is the smallest for the TEE method proposed in the present research (the smallest errors are highlighted and underlined).

The above-described concept will be further extended in the next section for the case of hybrid composites.

### 2.2. Effective Elastic Properties of Hybrid Composite Material

In a general case of a hybrid composite, the volume fraction of reinforcement (4) can be decomposed as follows:

(10) $$\xi =\frac{dV^{I_{1}}+\ldots +dV^{I_{n}}}{dV^{RVE}}=\sum_{i=1}^{n}\xi_{i}.$$

In engineering practice, the most common are hybrid composites made of two types of reinforcement $\left(I_{1},I_{2}\right)$ and one matrix material $\left(M\right)$. For such a three-phase isotropic composite with randomly oriented and distributed inclusions, the total fraction ξ of the reinforcement volume $dV^{I}$ in the total volume $dV^{RVE}$ of the RVE takes the form:

(11) $$\xi =\frac{dV^{I}}{dV^{RVE}}=\frac{dV^{I_{1}}+dV^{I_{2}}}{dV^{RVE}}=\xi_{1}+\xi_{2}.$$

In the case of a three-phase hybrid composite, the unknown functions $f_{1}\left(\xi_{1},\xi_{2}\right)$ and $f_{2}\left(\xi_{1},\xi_{2}\right)$ appearing in Equation (3) are therefore the functions of two variables, namely the volume fractions of both reinforcing materials $\xi_{1}$ and $\xi_{2}$. These functions must satisfy the boundary conditions at the two characteristic points:

(1) $\xi =\xi_{1}=\xi_{2}=0$ when a homogeneous pure matrix material is considered, therefore the stiffness tensor (7) becomes the stiffness tensor of the matrix material:
  (12) $$\xi_{1}=\xi_{2}=\xi =0\to E_{ijkl}\left(0,0\right)=E_{ijkl}^{M}\to \left\{\begin{array}{c} \lambda \left(0,0\right)=\lambda^{M}\\ \mu \left(0,0\right)=\mu^{M} \end{array}\right.\to \left\{\begin{array}{c} f_{1}\left(0,0\right)=0\\ f_{2}\left(0,0\right)=\frac{1}{2} \end{array}\right.,$$
(2) $\xi_{i}=1$ when the volume fraction of one of the reinforcement materials is equal to 1 (a homogeneous pure inclusion material is then considered). This second condition involves two cases: $\xi_{1}=1\wedge \xi_{2}=0$, and $\xi_{1}=0\wedge \xi_{2}=1$:
  (13) $$\xi_{i}=1\to \xi =1\to \left\{\begin{array}{l} \xi_{1}=1\wedge \xi_{2}=0\\ or\\ \xi_{1}=0\wedge \xi_{2}=1 \end{array}\right.\to \left\{\begin{array}{l} E_{ijkl}\left(1,0\right)=E_{ijkl}^{I_{1}}\\ or\\ E_{ijkl}\left(0,1\right)=E_{ijkl}^{I_{2}} \end{array}\right.\to \;\left\{\begin{array}{c} \lambda \left(1,0\right)=\lambda^{I_{1}}\wedge \mu \left(1,0\right)=\mu^{I_{1}}\\ \lambda \left(0,1\right)=\lambda^{I_{2}}\wedge \mu \left(0,1\right)=\mu^{I_{2}} \end{array}\right..$$

In the above expression ($\lambda^{I_{1}}$, $\mu^{I_{1}}$) and ($\lambda^{I_{2}}$, $\mu^{I_{2}}$) denote pairs of Lamé constants for the first and second inclusion material, respectively.

Taking into account expressions (7)–(9), the second boundary condition, (13), results in the following solutions for functions $f_{1}\left(\xi_{1},\xi_{2}\right)$ and $f_{2}\left(\xi_{1},\xi_{2}\right)$:

(14) $$f_{1}\left(\xi_{i}=1,0\right)=-\frac{1}{3}\left(\sqrt{\frac{\mu^{I_{i}}}{\mu^{M}}}-\sqrt{\frac{K^{I_{i}}}{K^{M}}}\right),\;f_{2}\left(\xi_{i}=1,0\right)=\frac{1}{2}\sqrt{\frac{\mu^{I_{i}}}{\mu^{M}}},\;i=1,2.$$

where $K^{M}$, $K^{I_{1}}$, and $K^{I_{2}}$ denote respectively the bulk moduli for matrix, inclusion-1, and inclusion-2 materials.

The first boundary condition therefore gives a single point solution:

(15) $$f_{1}\left(0,0\right)=0\wedge f_{2}\left(0,0\right)=\frac{1}{2}.$$

whereas two points result from the second boundary condition:

(16) $$f_{1}\left(1,0\right)=-\frac{1}{3}\left(\sqrt{\frac{\mu^{I_{1}}}{\mu^{M}}}-\sqrt{\frac{K^{I_{1}}}{K^{M}}}\right)\wedge f_{2}\left(1,0\right)=\frac{1}{2}\sqrt{\frac{\mu^{I_{1}}}{\mu^{M}}},$$

and

(17) $$f_{1}\left(0,1\right)=-\frac{1}{3}\left(\sqrt{\frac{\mu^{I_{2}}}{\mu^{M}}}-\sqrt{\frac{K^{I_{2}}}{K^{M}}}\right)\wedge f_{2}\left(0,1\right)=\frac{1}{2}\sqrt{\frac{\mu^{I_{2}}}{\mu^{M}}}.$$

The simplest linear approximation of the functions $f_{1}\left(\xi_{1},\xi_{2}\right)$ and $f_{2}\left(\xi_{1},\xi_{2}\right)$ between the boundary points (15), (16), and (17) has been here proposed:

(18) $$f_{1}\left(\xi_{1},\xi_{2}\right)=-\frac{1}{3}\left(\sqrt{\frac{\mu^{I_{1}}}{\mu^{M}}}-\sqrt{\frac{K^{I_{1}}}{K^{M}}}\right)\xi_{1}-\frac{1}{3}\left(\sqrt{\frac{\mu^{I_{2}}}{\mu^{M}}}-\sqrt{\frac{K^{I_{2}}}{K^{M}}}\right)\xi_{2},$$

(19) $$f_{2}\left(\xi_{1},\xi_{2}\right)=\frac{1}{2}\left(\sqrt{\frac{\mu^{I_{1}}}{\mu^{M}}}-1\right)\xi_{1}+\frac{1}{2}\left(\sqrt{\frac{\mu^{I_{2}}}{\mu^{M}}}-1\right)\xi_{2}+\frac{1}{2}.$$

The linear approximation between the boundary points together with the total strain energy equivalence hypothesis result in the second-degree polynomial description of the effective Lamé constants $\lambda \left(\xi_{1},\xi_{2}\right)$ and $\mu \left(\xi_{1},\xi_{2}\right)$ for the three-component hybrid composite. Taking into account (8)–(9) and (18)–(19) the following relations are finally obtained:

(20) $$\lambda \left(\xi_{1},\xi_{2}\right)=\lambda^{M}\left[{\left(C_{1}\xi_{1}+C_{2}\xi_{2}\right)}^{2}-2\left(C_{1}\xi_{1}+C_{2}\xi_{2}\right)\left(D_{1}\xi_{1}+D_{2}\xi_{2}+1\right)+{\left(D_{1}\xi_{1}+D_{2}\xi_{2}+1\right)}^{2}\right]\;+\frac{2}{3}\mu^{M}\left[{\left(C_{1}\xi_{1}+C_{2}\xi_{2}\right)}^{2}-2\left(C_{1}\xi_{1}+C_{2}\xi_{2}\right)\left(D_{1}\xi_{1}+D_{2}\xi_{2}+1\right)\right],$$

(21) $$\mu \left(\xi_{1},\xi_{2}\right)=\mu^{M}{\left(D_{1}\xi_{1}+D_{2}\xi_{2}+1\right)}^{2}.$$

where

(22) $$C_{i}=\sqrt{\frac{\mu^{I_{i}}}{\mu^{M}}}-\sqrt{\frac{K^{I_{i}}}{K^{M}}},\;\;\;\;\;\;\;\;i=1,2\;\;\;D_{i}=\sqrt{\frac{\mu^{I_{i}}}{\mu^{M}}}-1,\;\;\;\;\;\;\;\;\;\;\;\;\;\;\;i=1,2$$

The approach may be furtherly developed by replacing in (18)–(19) the linear functions with more advanced interpolations.

## 3. Results

### 3.1. Parametric Studies

It should be underlined that the proposed method does not involve any model parameters that need to be identified experimentally. Parametric studies in this case concern therefore only the analysis of the model response (prediction of effective composite properties) for different volume fractions of the fillers. To perform such parametric studies the hybrid composite material described in [32] was selected, which is composed of a polyamide matrix (PA) with the addition of aramid fibers (AF) and basalt fibers (BF). Aramid fibers are a kind of synthetic organic fibers with high fatigue and creep resistance. Basalt fibers are inorganic reinforcement characterized by high strength and stiffness. They have good chemical and high temperature resistance. The advantage is also their non-toxicity and environmental friendliness. Aramid fibers are here denoted as reinforcement material number 1 ($I_{1}$), while basalt fibers are named as filler number 2 ($I_{2}$). The Lamé constants and the bulk moduli for all the composite components are presented in Table 2.

**Figure 3:**
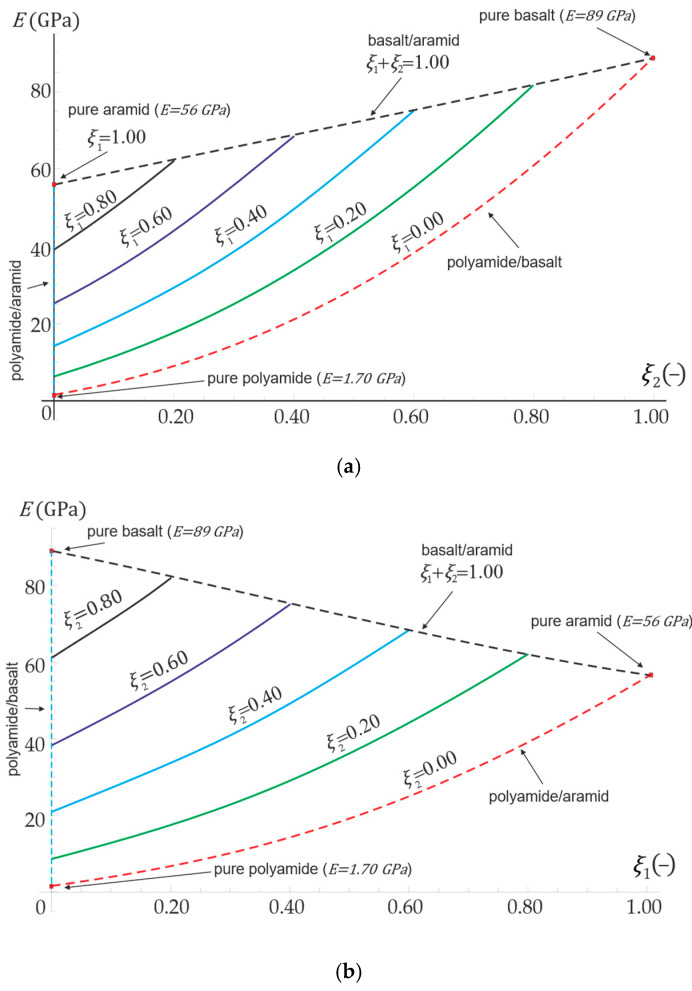
Distribution of effective Young modulus depending on the volume fraction of (**a**) basalt fibers $\left(\xi_{2}\right)$ with a constant content of aramid fibers $\left(\xi_{1}\right)$; (**b**) aramid fibers $\left(\xi_{1}\right)$ with a constant content of basalt fibers $\left(\xi_{2}\right)$.

Figure 3a shows the results assuming a constant volume fraction of the aramid fibers $\left(\xi_{1}\right)$ and a variable volume fraction of the basalt fibers $\left(\xi_{2}\right)$. Dashed lines indicate the limit cases when the volume fraction of one of the composite components (either matrix or one of the filaments) is zero, therefore a two-component material is then considered: polyamide/basalt at the lower curve, basalt/aramid at the upper line, and polyamide/aramid at the vertical dashed line. Also, at three characteristic points pure homogeneous material properties are properly reflected: $\xi_{1}=\xi_{2}=0$ (no fillers, pure polyamide matrix), $\xi_{1}=0$ and $\xi_{2}=1$ (no aramid fibers, no polyamide matrix, pure basalt), $\xi_{1}=1$ and $\xi_{2}=0$ (no basalt fibres, no polyamid matrix, pure aramid). Analogical simulations were performed for the constant volume fraction of the second reinforcement (basalt), while the content of the first one (aramid) is variable (Figure 3b). Similar conclusions may be drawn as for Figure 3a.

**Figure 4:**
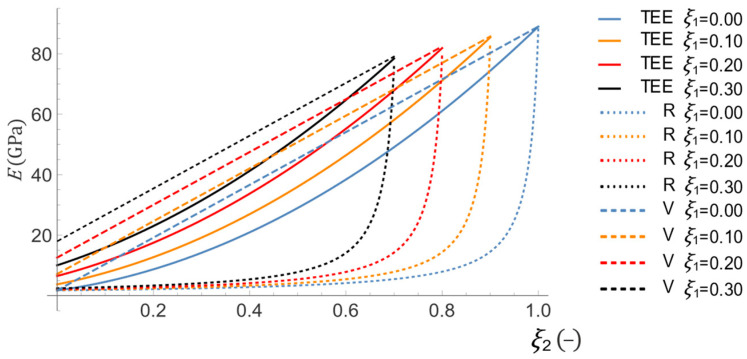
Comparison of effective Young modulus predictions depending on the volume fraction of basalt fibers $\left(\xi_{2}\right)$ with constant content of aramid fibers $\left(\xi_{1}\right)$ for total strain energy equivalence (TEE), Reuss (R), and Voigt (V) methods.

The obtained results were next compared with the Voigt and Reuss methods. Figure 4 shows the distribution of the effective Young modulus assuming a constant volume fraction of the aramid reinforcement $\left(\xi_{1}\right)$ and a variable volume fraction of the basalt reinforcement $\left(\xi_{2}\right)$. All the total strain energy equivalence-based predictions (TEE) are correctly placed between the lower (Reuss) and upper (Voigt) bounds.

### 3.2. Validation

Model validation was carried out for hybrid composites described in the available literature. The results for the (PA-AF-BF) composite described in the previous Section 3.1 will be discussed first. Table 3 contains the Young modulus and Poisson ratio for the components of this composite (see also Table 2).

**Figure 5:**
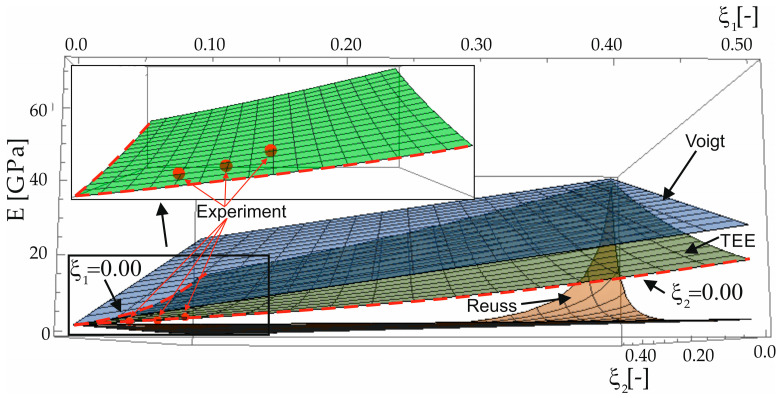
Distribution of effective Young modulus depending on the volume fraction of both types of fibers for the TEE, Reuss (R), and Voigt (V) methods.

**Figure 6:**
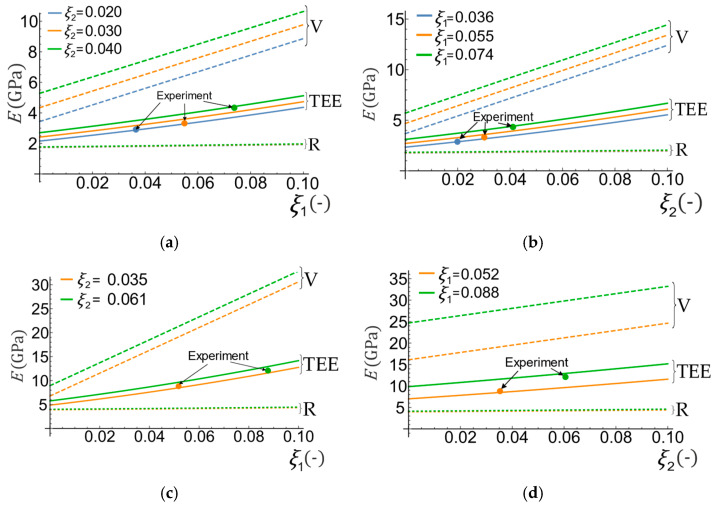
Comparison TEE, Reuss (R), and Voigt (V) methods with experimental results for: (**a**) polyamide reinforced with aramid fibers $\left(\xi_{1}\right)$. and basalt fibers $\left(\xi_{2}\right)$, variable volume fraction of aramid and constant volume fraction of basalt; (**b**) polyamide reinforced with aramid fibers $\left(\xi_{1}\right)$ and basalt fibers $\left(\xi_{2}\right)$, variable volume fraction of basalt and constant volume fraction of aramid; (**c**) polylactide reinforced with carbon fibers $\left(\xi_{1}\right)$ and basalt fibers $\left(\xi_{2}\right)$, variable volume fraction of carbon and constant volume fraction of basalt; (**d**) polylactide reinforced with carbon fibers $\left(\xi_{1}\right)$ and basalt fibers $\left(\xi_{2}\right)$, variable volume fraction of basalt and constant volume fraction of carbon.

The space distribution of the Young modulus $E\left(\xi_{1},\xi_{2}\right)$ as a function of two variables is shown in Figure 5. The 3D surface predicted by the proposed method (TEE) is shown juxtaposed with the Voigt and Reuss surfaces, and the available experimental data. In the enlarged fragment of the drawing, a very good agreement can be seen between the TEE predictions and the experimental data. Similar results are presented in Figure 6, where the experimental data are placed along with the lines created by the intersection of the surface $E\left(\xi_{1},\xi_{2}\right)$ with planes $\xi_{2}=const$ (variable volume fraction of aramid, constant volume fraction of basalt, Figure 6a) and $\xi_{1}=const$ (variable volume fraction of basalt, constant volume fraction of aramid, Figure 6b). 

The same validation was performed with the use of another hybrid composite material available in the literature, which is a polylactide (PLA) reinforced with carbon (CF) and basalt (BF) fibers [51]—see Figure 6c,d. In this case, the hybridization was aimed at producing a composite with comparable strength and stiffness as two-component PLA-BF composites, but with lower density, thanks to the synergistic combination of carbon and basalt fibers. Table 4 summarizes the material data for the components.

The validation results are summarized in Table 5. The best predictions (exhibiting the smallest error) are highlighted and underlined. In all the cases they appear to be the TEE method estimations.

## 4. Conclusions

The use of hybrid composites for semi-structural applications is increasing in many branches of industry. Hybrid composites are often more advantageous than classical two-component composites due to their environmental friendliness, weight reduction, and lower production costs. Structural applications of hybrid composites require knowledge of their mechanical properties, which result from the properties of constituent materials as well as their volume fractions and geometrical configuration. The commonly used estimation methods (for example the rule of mixtures) usually give results with a large error. For this reason, there is a need to develop a homogenization technique that would be accurate and preferably conceptually simple, not requiring complicated calculations.

The paper describes a new method of hybrid composite properties estimation. The approach is based on the mechanical equivalence between a real, heterogeneous material, composed of a matrix and reinforced inclusions, and a fictitious pseudo-homogeneous configuration. The fictitious pseudo-homogeneous material is mechanically equivalent to the real composite in the sense of the total internal strain energy being the same for both materials.

The proposed approach to properties estimation exhibits several important advantages in comparison with the most common homogenization methods. First of all, it has a physical, energy-based background. Additionally, the analysis is performed at the macro-scale, with the use of a mathematical formalism that does not require any assumptions about the exact size, type and distribution of reinforcing inclusions. The method can be used for an arbitrary number of composite components without any further simplifications or the need for multi-step calculations. The predictions are very close to the experimental values, which confirms that the physical assumptions of the approach are correct.

One of the most important features of the proposed estimation method is its simplicity: there is no need to numerically homogenize the composite components. What is more, the estimates are given by the smooth analytical functions of the component material properties, while there is no need to identify any additional parameters. Such functions are convenient for example for optimization problems.

In the present paper, the method was validated for isotropic short-fiber reinforced hybrid composites in the elastic range of response. However, it is possible to extend the approach to anisotropic materials. In that case instead of a scalar volume fraction parameter ξ, a tensorial quantity should be utilized, which can reflect not only the fraction of volume but also the geometrical configuration of inclusions. The proposed approach can also be applied to inelastic composite parameters estimations due to the contribution of both elastic and inelastic strains to the total strain energy. 

Finally, it should be pointed out that the proposed approach does not take into account some of the conditions that affect the effective properties of hybrid composites. For example, the influence of the reinforcing particle size is here disregarded, as well as the (surface) varying properties of interphases.

## Figures and Tables

**Table 1:** Comparison of estimation errors for data from Figure 2.

Composite	ξ (-)	Experimental Young Modulus E (GPa)	Estimation Method Error (%)					
			Energy Equivalence Based Method (TEE)	Voigt (V)	Reuss (R)	Hashin- Shtrikman Upper (HSU)	Hashin- Shtrikman Lower (HSL)	Mori-Tanaka (MT)
POM-CF	0.00	3.42	0.00	0.00	0.00	0.00	0.00	0.00
	0.05	7.03	10.31	107.82	48.83	32.64	46.17	46.17
	0.10	9.64	4.39	167.53	60.65	60.45	56.57	56.57
	0.20	16.58	21.83	190.39	74.31	73.20	69.03	69.03
	0.30	24.00	40.94	193.77	79.77	81.17	73.58	73.58
PA-GF	0.05	4.04	13.14	69.78	16.82	28.26	12.59	12.59
	0.07	5.08	2.03	63.07	32.49	17.84	27.70	27.70
	0.13	7.77	6.89	60.81	52.97	8.92	46.83	46.83
	0.16	8.57	2.22	70.12	55.92	13.70	48.87	48.87
	0.23	11.63	2.31	66.92	64.73	10.64	56.72	56.72
	0.31	13.06	17.09	90.73	65.18	27.91	54.70	54.70
PE-HAp	0.00	1.30	0.00	0.00	0.00	0.00	0.00	0.00
	0.10	1.40	49.99	110.34	2.25	59.09	11.73	11.73
	0.20	2.00	53.18	124.38	20.37	60.58	5.98	5.98
	0.25	2.50	44.16	109.51	32.50	49.26	17.47	17.47
	0.30	3.00	39.41	99.28	40.18	42.44	24.46	24.46
	0.35	3.70	29.66	81.39	48.22	30.79	32.63	32.63
	0.40	4.40	23.84	69.07	53.29	23.45	37.55	37.55
	0.45	5.90	4.00	38.33	62.44	2.61	48.54	48.54
	0.50	7.70	10.93	15.33	68.78	12.88	56.28	56.28

**Table 2:** Material properties of composite components.

Material	Bulk Modulus K (GPa)	Lamé Constant λ (GPa)	Lamé Constant μ (GPa)
PA	2.36	1.95	0.62
AF	49.12	34.87	21.37
BF	61.81	38.26	35.32

**Table 3:** Material properties of matrix polyamide, aramid fibers, and basalt fibers (data taken from [32]).

Material	Young Modulus E (GPa)	Poisson Ratio ν (-)
PA	1.70	0.38
AF	56.00	0.31
BF	89.00	0.26

**Table 4:** Material properties of matrix polylactide, carbon fibers, and basalt fibers (data taken from [32,51,52,53]).

Material	Young Modulus E (GPa)	Poisson Ratio ν (-)
PLA	3.76	0.36
CF	242.00	0.32
BF	89.00	0.26

**Table 5:** Comparison of numerical values and estimation errors for data from Figure 5 and Figure 6.

Composite	$\xi_{1}(-)$	$\xi_{2}\;(-)$	Experimental E (GPa)	Estimation Method					
				TEE		Voigt (V)		Reuss (R)	
				Value E (GPa)	Error (%)	Value E (GPa)	Error (%)	Value E (GPa)	Error (%)
PA-AF-BF	0.036	0.020	2.89	2.87	0.61	5.41	87.19	1.80	37.77
	0.055	0.030	3.31	3.59	8.61	7.33	121.40	1.85	43.99
	0.074	0.040	4.32	4.42	2.22	9.29	115.07	1.91	55.69
PLA-CF-BF	0.052	0.035	8.84	8.49	3.93	19.12	116.27	4.11	53.52
	0.088	0.061	12.13	12.93	6.62	29.85	146.12	4.40	63.77

## Data Availability

Not applicable.
